# Supplementary material for: Variant phasing and haplotypic expression from long-read sequencing in maize
Source: Commun Biol. 2020 Feb 18;3:78. doi: 10.1038/s42003-020-0805-8 (PMC7028979; doi:10.1038/s42003-020-0805-8)
Supplement: Supplementary file 1 — Supplementary Information [file 42003_2020_805_MOESM1_ESM.pdf]

Supplementary Figures

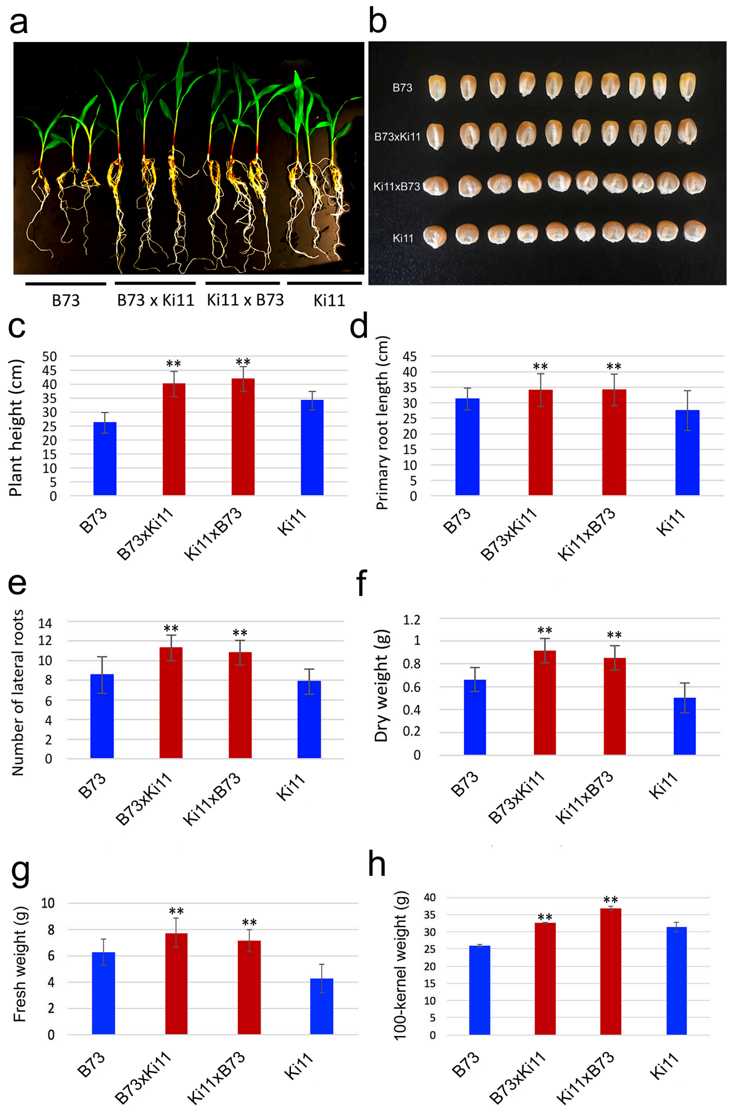

Supplementary Figure 1. Phenotype of maize B73, Ki11, and two reciprocal hybrids (B73 × Ki11, Ki11 × B73) at 14DAG and measure of different traits between parents and the hybrids. (a) Shoot and root phenotype of B73, Ki11, and the two reciprocal hybrids. (b) Seed phenotype of B73, Ki11, and the two hybrids. (c) Plant height, (d) primary root length, (e) lateral root number, (f–g) biomass, and (h) 100-kernel weight of the parents and hybrids. ** p<0.01. Error bars are S.D. on the three biological replicates, 10 biologically independent plants were used for each replicate.

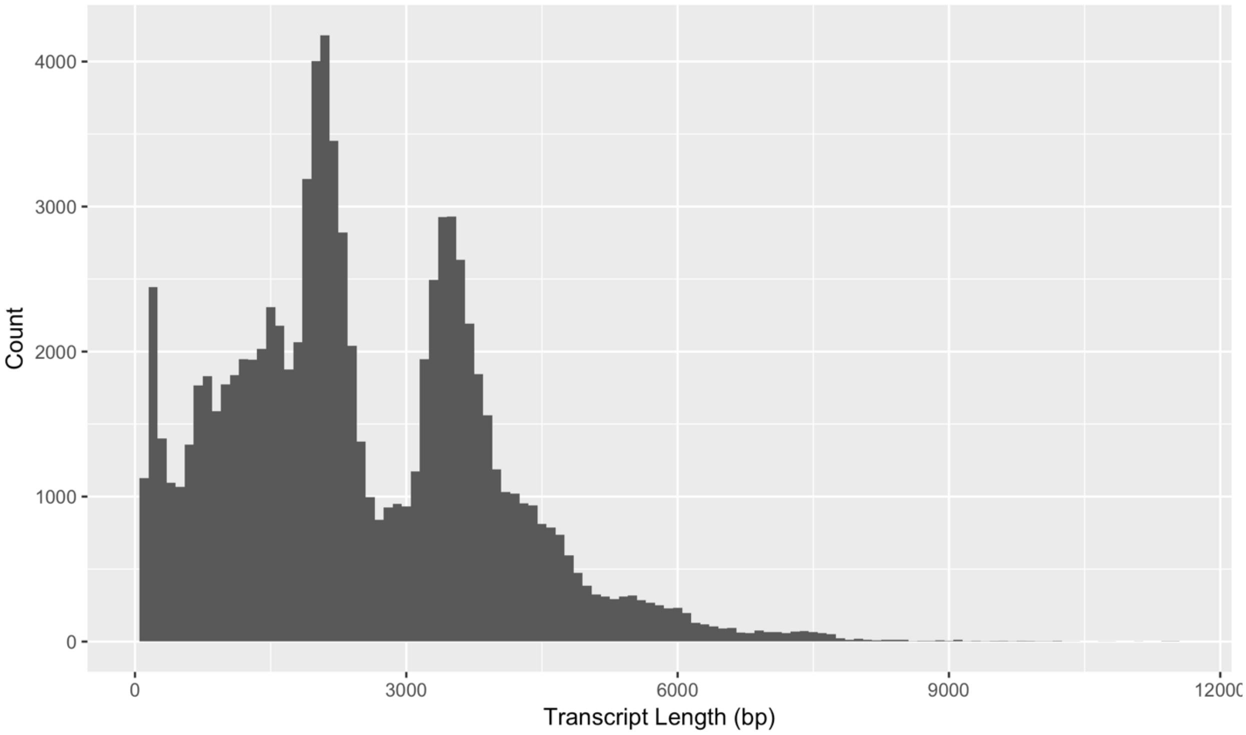

Supplementary Figure 2. Length distribution of the final transcript set.

After mapping the high-quality (HQ) sequences to the B73 RefGen_v4 genome and filtering for coverage, identity, and running the SQANTI software to remove library artifacts, we obtain 75,118 transcripts. Min: 80bp, Max: 11,495 bp, Mean: 2,482 bp, 5th-95th percentile: 363-4,975 bp.

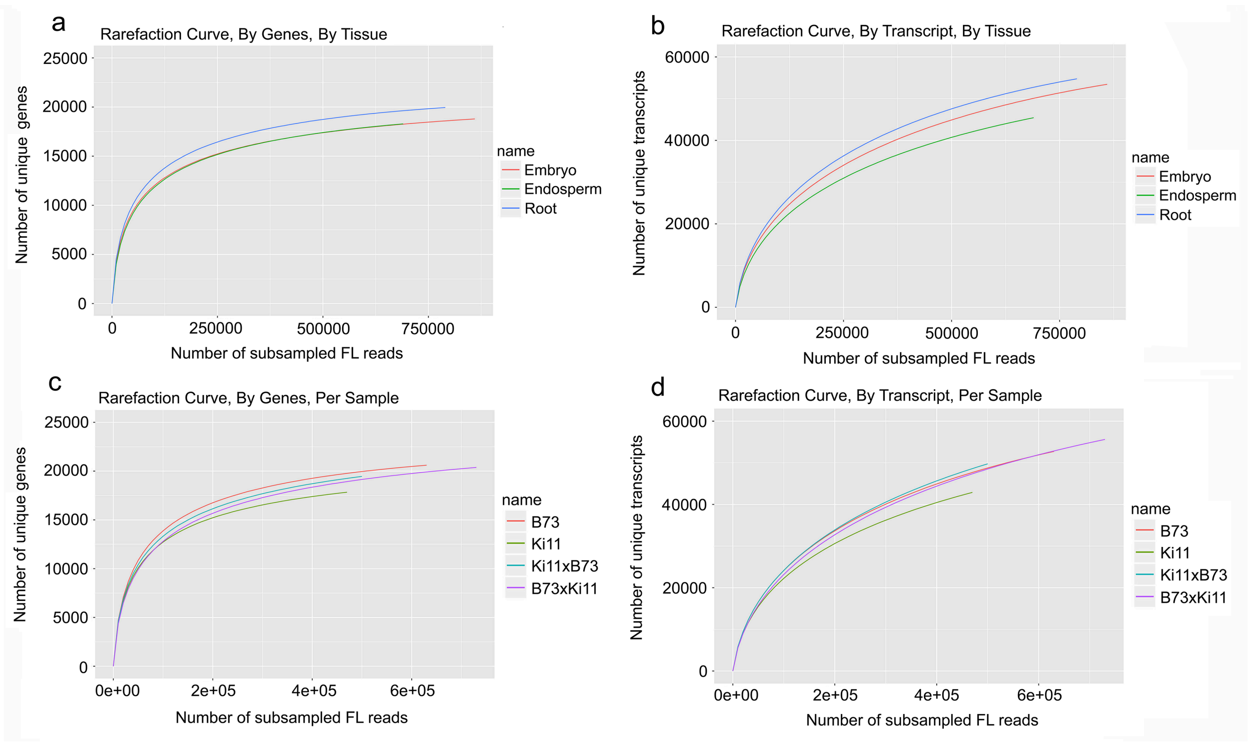

Supplementary Figure 3. Rarefaction curves against known genes and transcripts (a-b) by strain, (c-d) by tissue. For each subpanel, the X-axis shows the number of subsampled full-length reads and the Y-axis shows the number of observed unique genes or transcripts. Both known and novel genes/transcripts are considered.

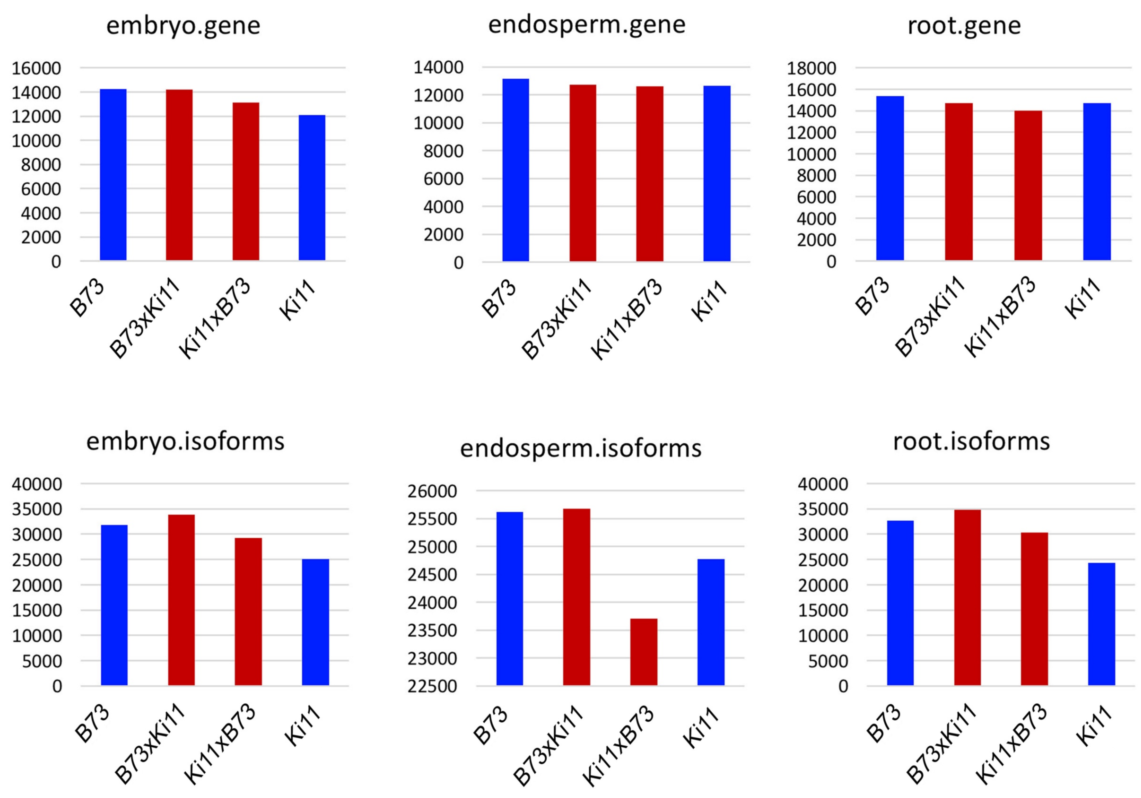

Supplementary Figure 4. Number of genes and isoforms between parents and two reciprocal hybrids in embryo, endosperm and root tissues.

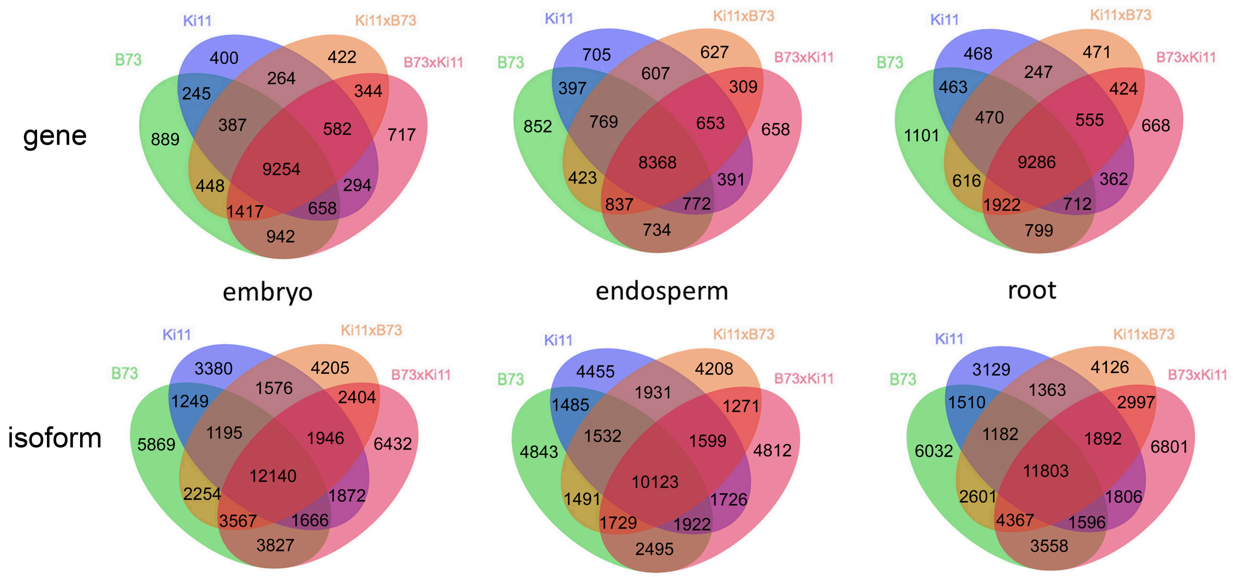

Supplementary Figure 5. Overlap of genes and isoforms among parents and two hybrids in embryo, endosperm and root.

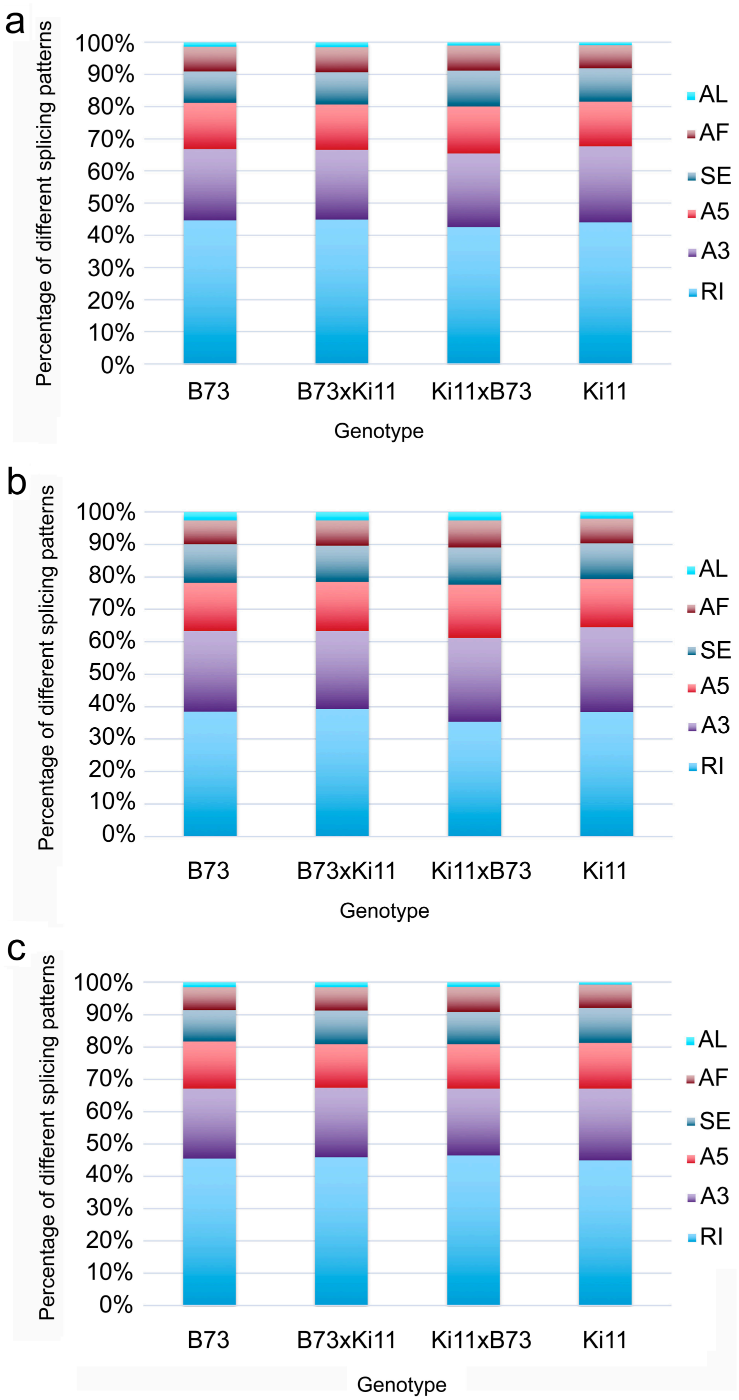

Supplementary Figure 6. Alternative splicing pattern between parents and two reciprocal hybrids in embryo (a), endosperm (b) and root (c). A3: Alternative 3' splice site; A5: Alternative 5' splice site; AF: Alternative first exon' AL: Alternative last exon; RI: Retained Intron; SE: Skipped exon.

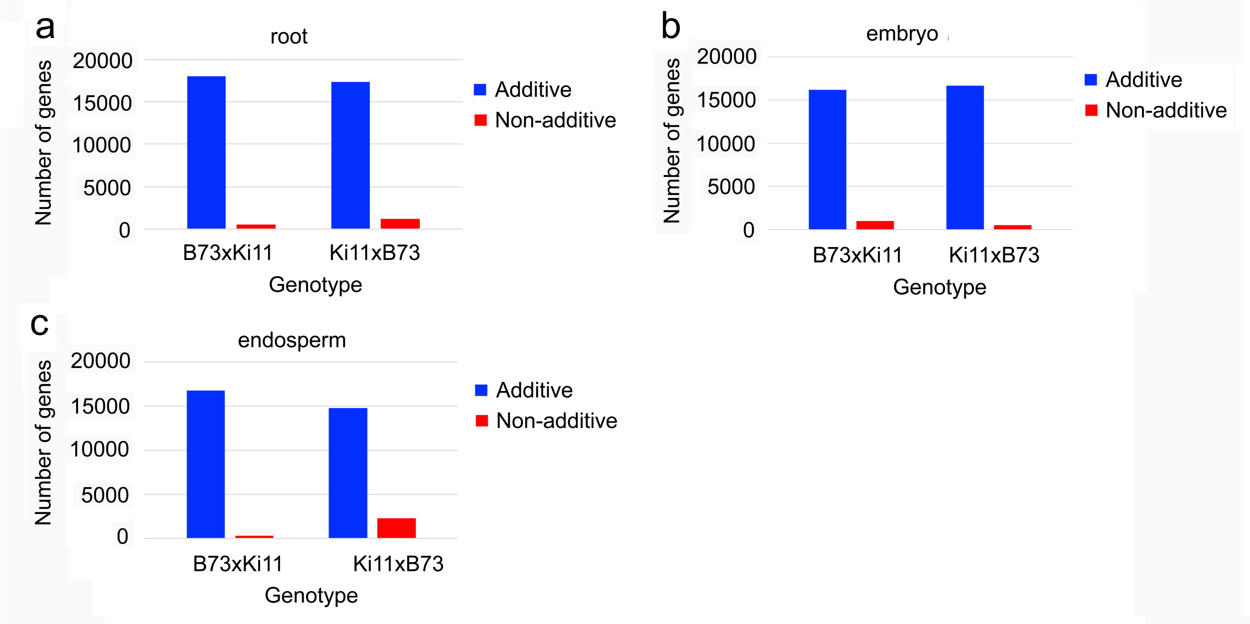

Supplementary Figure 7. Number of additive and non-additive expression genes in root (a), embryo (b) and endosperm (c).

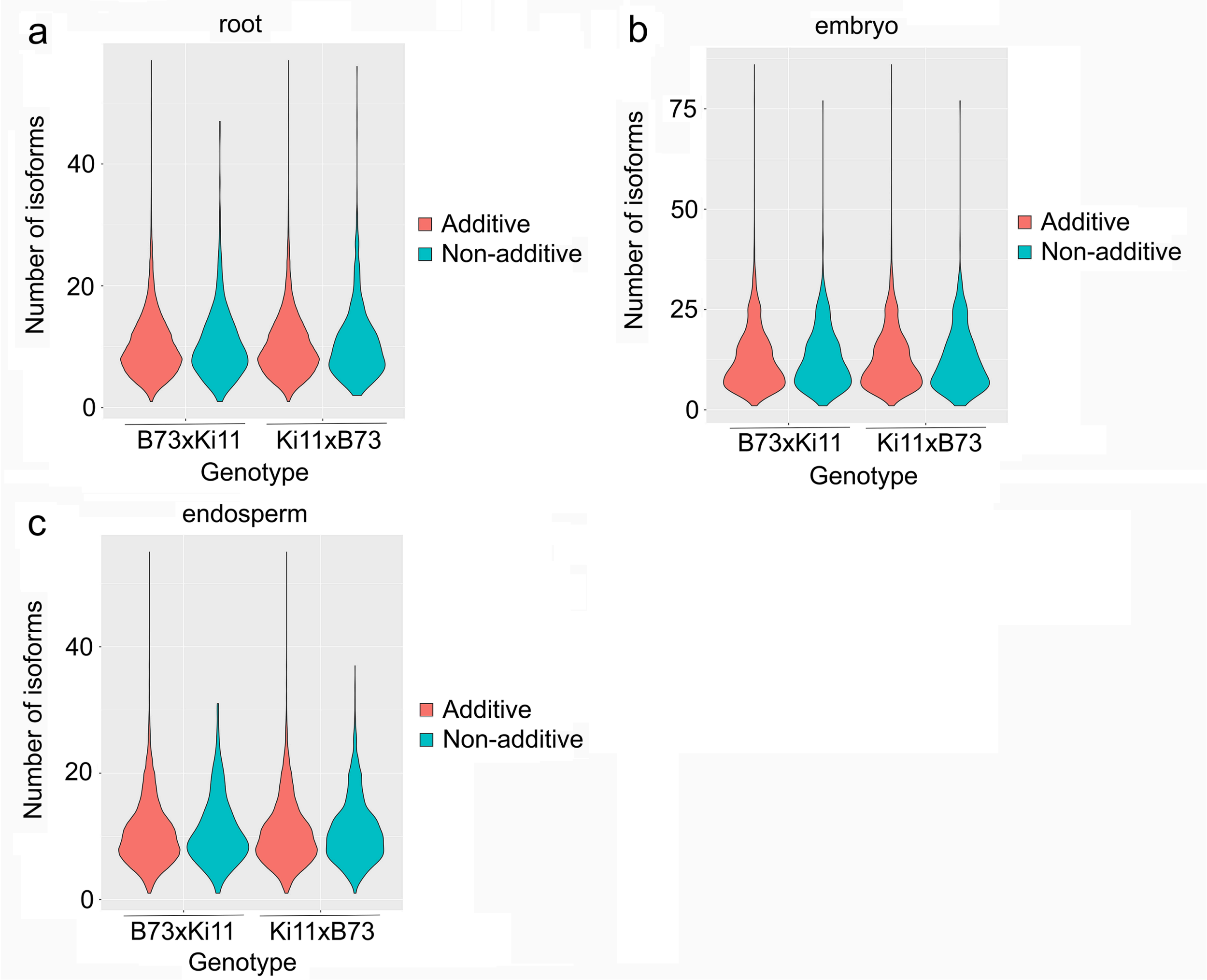

Supplementary Figure 8. Number of isoforms of additive and non-additive expression genes in root (a), embryo (b) and endosperm (c).

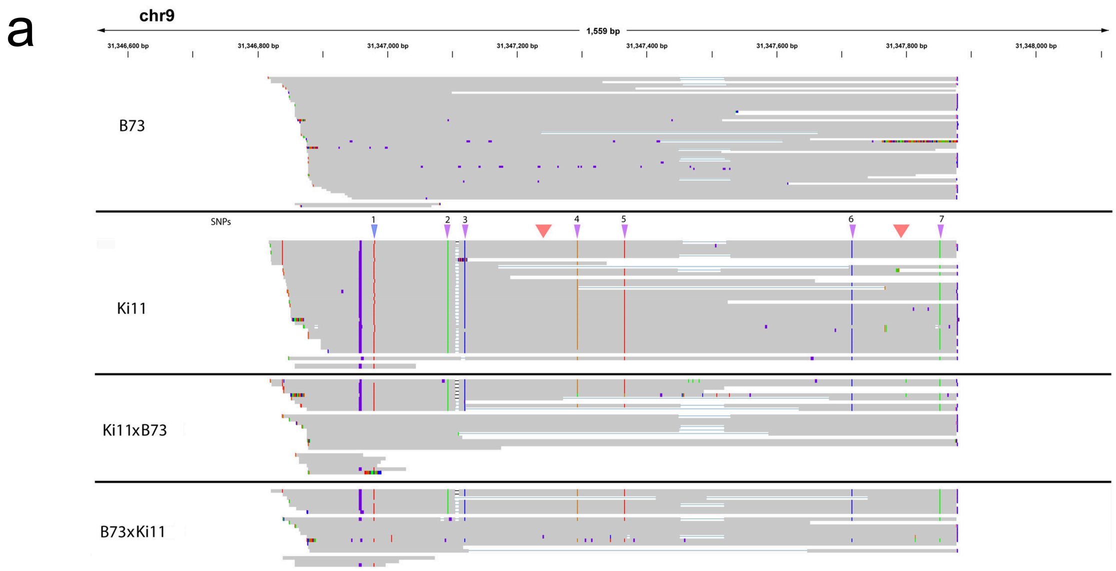

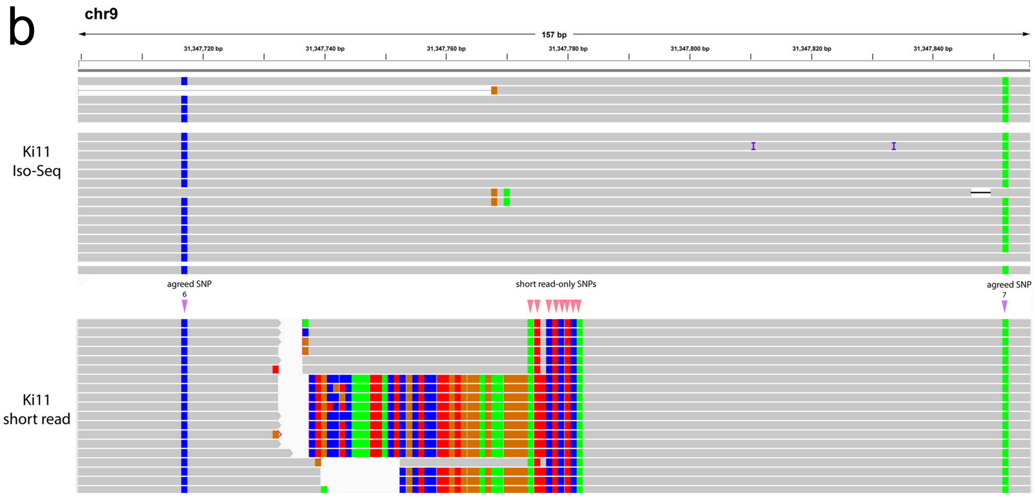

Supplementary Figure 9. IsoPhase phasing example. a) The gene PB.21897 (Zm00001d045657) phased by IsoPhase. SNPs are depicted between the B73 and Ki11 tracks, seven SNPs are shown. SNP #2–#7 were called by based on both long- and short-read data (purple), SNP #1 was missed by long-read data due to reduced coverage (blue); suspicious short read-only SNPs are marked in red. b) Zoomed-in region between SNP #6 and SNP #7 showing suspicious SNPs (red) called based on short-read only. Top track shows Ki11 Iso-Seq FL reads; bottom track shows Ki11 short reads.

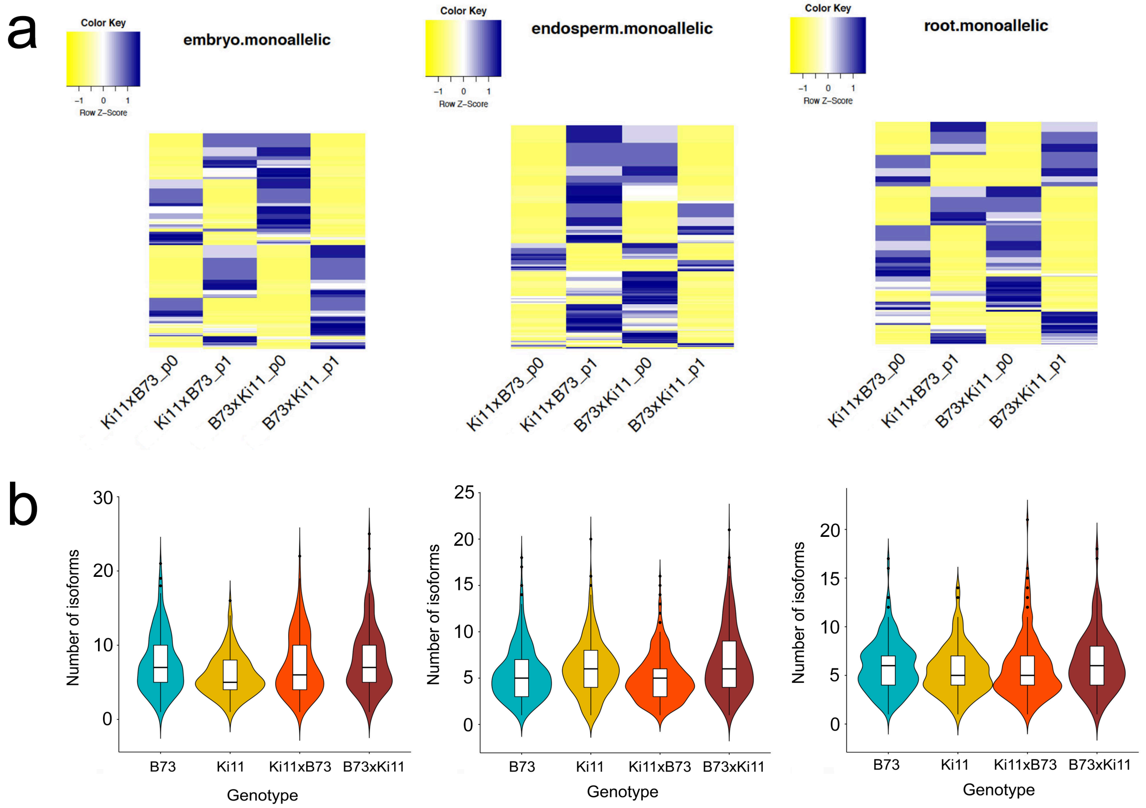

Supplementary Figure 10. Expression and number of isoforms of mono-allelic genes in different tissues. a) Allelic expression of mono-allelic genes in reciprocal hybrids, and b) number of isoforms between parents and hybrids of mono-allelic genes. p0 represents the B73 allele; p1 represents the Ki11 allele.

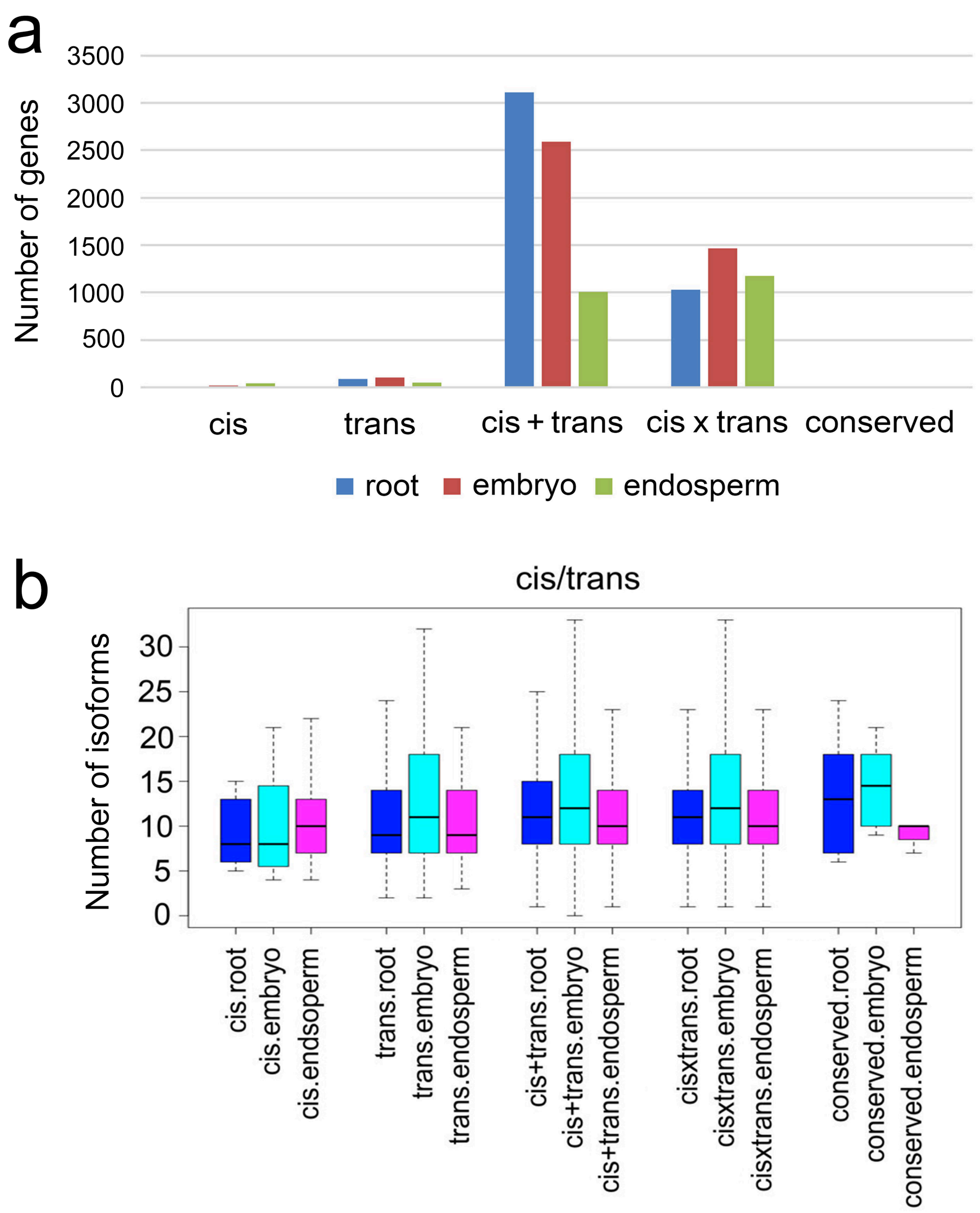

Supplementary Figure 11. Distribution of cis-, trans-regulated genes, and number of isoforms of each category. (a) Distribution of different categories of cis-, trans-regulated genes. (b) Number of isoforms in each category of cis-, trans-regulated genes.

Supplementary Table 1. 16-mer Barcodes corresponding to the Sample.

Sample	Genotype	Barcode Sequence
Embryo 1	B73 20 DAP	TCAGACGATGCGTCAT
Embryo 2	Ki11 20 DAP	TACTAGAGTAGCACTC
Embryo 3	Ki11xB73 20 DAP	GATCTCTACTATATGC
Embryo 4	B73xKi11 20 DAP	CATAGCGACTATCGTG
Endosperm 1	B73 20 DAP	CTATACATGACTCTGC
Endosperm 2	Ki11 20 DAP	TGTGTATCAGTACATG
Endosperm 3	Ki11xB73 20 DAP	ACAGTCTATACTGCTG
Endosperm 4	B73xKi11 20 DAP	CGAGCACGCGCGTGTG
Root 1	B73 20 DAP	GCTCGACTGTGAGAGA
Root 2	Ki11 20 DAP	TGCTCGCAGTATCACA
Root 3	Ki11xB73 20 DAP	TCACACTCTAGAGCGA
Root 4	B73xKi11 20 DAP	CGCTGCGAGAGACAGT

Supplementary Table 2. Number of full-length, non-concatemer (FLNC) reads from each of the 12 samples after demultiplexing.

SAMPLE	NAME	# of FLNC
EM1	B73 Embryo	339,048
EM2	Ki11 Embryo	254,342
EM3	Ki11xB73 Embryo	305,307
EM4	B73xKi11 Embryo	444,580
END1	B73 Endosperm	284,678
END2	Ki11 Endosperm	290,122
END3	Ki11xB73 Endosperm	232,168
END4	B73xKi11 Endosperm	288,205
R1	B73 Root	362,431
R2	Ki11 Root	225,208
R3	Ki11xB73 Root	287,485
R4	B73xKi11 Root	426,238
**TOTAL**		**3,739,812**

Supplementary Table 3. Mapping high-quality (HQ) transcript sequences to the genome and filtering criteria. HQ sequences were mapped to B73 v4 genome and filtered for 99% coverage and 95% identity. Redundant transcripts are collapsed.

**Mapping to Genome**	
HQ Isoforms	250,168
Mapped to maize v4	248,424 (99.3%)
Mapped to maize v4 with 99% coverage 95% identity	229,757 (91.8%)
**After collapse & compare to annotation:**	
Number of unique isoforms	90,419<br>(27,967 loci)

Supplementary Table 4. Removal of library artifacts using a modified version of the SQANTI software.

Cause of SQANTI Filtering	Transcripts Filtered
**BEFORE FILTERING:**	90,419 transcripts<br>(27,967 loci)
Intrapriming	1402
RT-switching	1906
Some junctions not canonical and not all junctions short-read-supported	11993
**AFTER FILTERING:**	75,118 transcripts<br>(23,412 loci)

Supplementary Table 5. Top BLASTN hit counts of the unmapped high-quality (HQ) transcript sequences to the NR database. BLASTN was run with a report of best hit with E-value cutoff of 0.1; 1669 of 1744 HQ sequences had a BLASTN hit.

Species	Sequence_Hit_Counts
Bipolaris zeicola	534
Zea mays	523
Cochliobolus sativus	200
Fusarium verticillioides	162
Bipolaris maydis	63
Trichoderma virens	39
Bipolaris victoriae	17
Setosphaeria turcica	9

Supplementary Table 6. Sample-specific transcript counts. Using the demultiplexed full-length reads, we assigned Iso-Seq transcripts back to each sample. If a transcript contained at least one full-length read from a sample, it was considered to be expressed.

Sample	Number
B73-Embryo	31,767
B73-Endosperm	25,620
B73-Root	32,649
Ki11-Embryo	25,024
Ki11-Endosperm	24,773
Ki11-Root	24,281
Ki11xB73-Embryo	29,287
Ki11xB73-Endosperm	23,704
Ki11xB73-Root	30,331
B73xKi11-Embryo	33,854
B73xKi11-Endosperm	25,677
B73xKi11-Root	34,820

Supplementary Table 7. Fleiss' kappa of gene-level agreement and isoform-level agreement in embryo, endosperm, and root tissue between long and short reads.

Tissue	Gene	Isoform
embryo	0.905	0.958
endosperm	0.915	0.964
root	0.926	0.966

Genes and isoforms were assigned to groups of genotypes according to whether they were expressed in each tissue. These assignments were compared to the long-read data assignments using Fleiss' kappa, a statistical measure that calculates the degree of agreement in classification over what would be expected by chance.
