# Supplementary material for: Variant phasing and haplotypic expression from long-read sequencing in maize
Source: Commun Biol. 2020 Feb 18;3:78. doi: 10.1038/s42003-020-0805-8 (PMC7028979; doi:10.1038/s42003-020-0805-8)
Supplement: Supplementary file 2 — Description of Additional Supplementary Files [file 42003_2020_805_MOESM2_ESM.docx]

**Description of Supplementary Data**

**Supplementary Data 1.** Full-length read count three tissues in each haplotype of each genotype, as determined by IsoPhase.

**Supplementary Data 2.** Full-length and short-read counts of imprinted genes in endosperm and embryo tissues.

**Supplementary Data 3.** List of imprinted genes in 20DAP endosperm and embryo.
